# Mediating Effect of Burnout on the Association between Work-Related Quality of Life and Mental Health Symptoms

**DOI:** 10.3390/brainsci11060813

**Published:** 2021-06-19

**Authors:** Henrique Pereira, Gergely Feher, Antal Tibold, Vítor Costa, Samuel Monteiro, Graça Esgalhado

**Affiliations:** 1 Department of Psychology and Education, Faculty of Social and Human Sciences, University of Beira Interior, Pólo IV, 6200-209 Covilhã, Portugal; vitormvc@ubi.pt (V.C.); smonteiro@ubi.pt (S.M.); mgpe@ubi.pt (G.E.); 2 Research Centre in Sports Sciences, Health Sciences and Human Development (CIDESD), 5001-801 Vila Real, Portugal; 3 Centre for Occupational Medicine, Medical School, University of Pécs, 7624 Pécs, Hungary; feher.gergely@pte.hu (G.F.); tibold.antal@pte.hu (A.T.); 4 NECE—Research Center in Business Science, University of Beira Interior, Pólo IV, 6200-209 Covilhã, Portugal; 5 Institute of Cognitive Psychology, Human and Social Development (IPCDHS), 3000-115 Coimbra, Portugal

**Keywords:** burnout, work-related quality of life, mental health, depression, anxiety, somatization

## Abstract

The purpose of this study was: (1) to assess levels of burnout, work-related quality of life (WRQoL) and mental health symptoms among a sample of active workers living in Portugal; (2) to analyze differences in burnout, WRQoL and mental health symptoms by gender and shift work; (3) to analyze association levels among all variables under study; (4) to determine the predictive effect of burnout and WRQoL on mental health symptoms; and (5) to assess the mediating effect of burnout on the association between WRQoL and mental health symptoms. Eight-hundred and forty-one Portuguese active workers between 18 and 67 years of age participated in this study (Mean = 37.23; SD = 11.99). Results showed that women participants scored higher in burnout and mental health symptoms, and lower in overall WRQoL, than men; additionally, participants who worked in shifts presented higher mental health symptoms. Significant correlations were found for all variables and regression analysis demonstrated that 56% of the overall variance of mental health symptoms was explained by older age, shift work, lower WRQoL, and burnout (exhaustion and cognitive impairment). Finally, the mediation effect of burnout on the association between WRQoL and mental health symptoms was statistically significant. These findings are useful for health professionals and health managers who work in the field of occupational health in identifying variables affecting burnout, WRQoL and mental health symptoms.

## 1. Introduction

Burnout, depression and anxiety symptoms are common mental health problems [1], with severe productivity costs for organizations due to impaired work performance [2]. Therefore, understanding the antecedents of workers’ mental health symptoms and the role of burnout and perceived work-related quality of life (WRQoL) is an important research topic. The present research examines occupational mental health hazards, analyzing burnout’s mediator role as a consequence of experiences in working life and as a phenomenon that may have potential after-effects on mental health. Hence, we describe and analyze the potential impact of occupational quality of life on mental health indicators, contributing to a better understanding of burnout syndrome predictors and health consequences.

### 1.1. Burnout—Conceptualization/Assessment

Burnout is an intricate, multi-causal process that involves various factors at different levels [3]. As a potential mediator variable in a relationship between WRQoL and mental health (anxiety, depression, and somatization symptom indicators), this research expands the use of a “novel” conceptualization of burnout.

The hegemonic and almost exclusive use of the Maslach Burnout Inventory (MBI) instrument on burnout assessment/research has negatively influenced its theoretical delimitation [4,5]. Traditionally, burnout was a three-dimensional phenomenon (i.e., emotional exhaustion, depersonalization/cynicism, and reduced personal accomplishment) [6], but the MBI assessment could not give an accurate and integrated perspective on burnout. Alternatively, the Burnout Assessment Tool (BAT), used in this research, offers a step forward on previous burnout domain research [5].

Burnout from a BAT assessment perspective is a severe work-related state of exhaustion characterized by extreme tiredness, dysfunctional and impaired capacity for regulating emotional and cognitive processes, and mental distancing [7]. In this research approach, emotional impairment and cognitive impairment are the central components of burnout syndrome and may last for a long time, even after other burnout symptoms have diminished. Thus, exhaustion and mental distancing may be considered the core dimensions of burnout [3]. Depressed mood and non-specific psychological and psychosomatic distress symptoms could also articulate the previous core symptoms and could be considered secondary burnout symptoms [7]. Variables interrelated to burnout are more evident and described in current research, but many remain to be understood, particularly in the variable interrelationships. This research adopts an exploration of predictors and consequent variables of burnout syndrome, also testing its possible mediating role.

**Figure 1:**
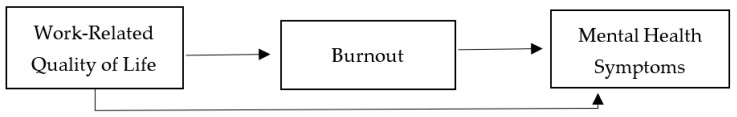
Conceptual model and main variables.

From a theoretical perspective, following the Job Demand-Resources (JD-R) model, burnout is the product of an interaction between demands/resources; it is not directly and objectively analyzed, but considered through a perceptive occupational indicator of the individual/occupation interaction quality, i.e., WRQoL (cf. Figure 1).

In terms of potential consequences, in this paper, we considered exclusively the deterioration process (provided in a branch of the JD-R model) expected and sought to analyze the potential consequences and impact of burnout on health (negative symptoms). In this way, we aimed to expand the use and the applied research of a “novel” burnout conceptualization as a potential mediator variable in a relationship between WRQoL and mental health indicators (anxiety, depression, and somatization symptoms).

### 1.2. WRQoL Implications and Burnout

Work-Related Quality of Life (WRQoL) is a broad concept used to define the quality of life of individuals at their workplace, be it of any type or size [8]. The literature on the topic has focused on aspects ranging from job satisfaction and work commitment to non-work life aspects, such as work-family issues [9]. The different conceptualizations have translated into multiple dimensions of the construct and measurement instruments that can be found in the literature [8,10,11,12]. In an insightful work, Van Laar et al. [13] argue that WRQoL should include work and non-work factors when the latter affects how the employee approaches work and is influenced by it. The cited authors developed a WRQoL tool that encompasses the following dimensions: job and career satisfaction, general well-being, home–work interface, stress at work, control at work and working conditions.

Therefore, WRQoL encompasses dimensions that significantly overlap with variables that have been extensively studied in their association with burnout, such as work stress and job satisfaction [14,15] or work–family conflict [16]. An increased WRQoL has been related to positive outcomes such as enhanced work engagement [17], occupational commitment [18] and life satisfaction [19]. Studies by Tuuli and Karisalmi [20] and Cetrano et al. [21] have shown the impact of WRQoL on burnout. More recent studies have found a strong and significant negative correlation between WRQoL and job burnout [22], with WRQoL being a strong negative predictor of job burnout [23].

### 1.3. Burnout Consequences and Mental Health Symptoms

As a syndrome, burnout is one of the most common chronic and work-related health complaints; today, it is increasingly experienced by people [24] as a result of prolonged interpersonal stressors at work [25]. Burnout is related to several work-related problems, such as absenteeism and intention to leave the job [26,27], and reduced performance in the workplace [28]. Additionally, inability and unwillingness to spend effort at work are the core effects of burnout [7] and may impact a person’s occupational performance and well-being, with potential consequences on productivity [29]. Hence, burnout is a mediator of the relationship between job demands and health problems [30]; many studies have also shown that it has negative impacts on workers’ physical and mental well-being [31,32]. In fact, it is possible that those who suffer from burnout have been diagnosed with a depressive and/or anxiety disorder [33].

Whether burnout is a form of depression and/or anxiety is an object of scientific controversy. Some burnout symptoms appear to resemble those of depression (e.g., loss of interest or pleasure, depressed mood, fatigue or loss of energy, impaired concentration, feelings of worthlessness, decreased or increased appetite, sleep problems, suicidal ideation) [34], but a causal relationship between burnout and depression symptoms may also exist [35]. Furthermore, burnout symptoms can also resemble those of anxiety, resulting in psychological distress and affecting a person’s everyday functioning [36,37].

### 1.4. Objectives

Studies conducted in Portugal exploring the mediating effect of the association between WRQoL and mental health symptoms are still scarce. Therefore, the purpose of this study was: (1) to assess levels of burnout, WRQoL and mental health symptoms among a sample of active workers living in Portugal; (2) to analyze differences in burnout, WRQoL and mental health symptoms by gender and shift work; (3) to analyze association levels among all variables under study; (4) to determine the predictive effect of burnout and WRQoL on mental health symptoms; and (5) to assess the mediating effect of burnout on the association between WRQoL and mental health symptoms.

## 2. Materials and Methods

### 2.1. Measurement Instruments

Burnout. The Portuguese version of the BAT was used in this study [7]. The BAT includes 22 items, measuring four core symptoms of burnout: exhaustion (eight items; e.g., “At work, I feel mentally exhausted,” α = 0.89), mental distance (four items; e.g., “I struggle to find any enthusiasm for my work,” α = 0.83), emotional impairment (five items; e.g., “At work, I feel unable to control my emotions,” α = 0.81), and cognitive impairment (five items; e.g., “At work, I have trouble staying focused,” α = 0.86). Alternatively, assuming that burnout is a syndrome, the BAT instrument enables an integrated perspective. This means that all four dimensions are interrelated and refer to the same underlying condition [38]. All items were scored on a five-point Likert scale ranging from 1 (never) to 5 (always). Responses were averaged for each subscale. To determine overall internal reliability, a Cronbach’s α = 0.92 was obtained, demonstrating excellent internal reliability.

Work-Related Quality of Life. The study utilized the Portuguese version of the Work-Related Quality of Life scale [39] to assess WRQoL. This is a Likert-type scale with responses ranging from 1 to 5, and it encompasses 23 items distributed across six dimensions, comprised of general well-being, home–work interactions, career satisfaction, control over work, working conditions, and work-related stress. For the purposes of this study, we used one overall measure of WRQoL resulting from the averaged responses for each subscale. To determine overall internal reliability, a Cronbach’s α = 0.91 was obtained, demonstrating excellent internal reliability.

Mental Health Symptoms. This study utilized the Portuguese version of the Brief Symptom Inventory-18 (BSI-18) [40] to measure participants’ mental health symptoms. The BSI-18 is comprised of 18 items that evaluate the psycho symptomatology experienced by individuals, using three subscales that examine somatization, depression and anxiety symptoms. The depression subscale focuses on the core symptoms of depressive disorders, which include dysphoric mood states and anhedonia, among others. In turn, the anxiety subscale encompasses symptoms indicative of panic states, such as nervousness, tension, and motor agitation. The global severity index is obtained by adding the scale’s 18 items together, providing a measurement of individuals’ general psychological distress with higher scores revealing more intense psycho symptomatology. All items were scored on a five-point Likert scale ranging from 0 (never) to 4 (always). To determine overall internal reliability, a Cronbach’s α = 0.93 was obtained, demonstrating excellent internal reliability.

### 2.2. Procedures

This research was carried out through a website that was available between October and December 2020. Participation was voluntary and participants were referred to a questionnaire on a website created specifically for the purpose of this investigation. The first page of the questionnaire explained the objectives of the study and informed participants about how to fill it out, how to withdraw from the study, and how to contact the authors for more information. They were also asked to read and agree to an informed consent waiver.

A total of about 4950 notifications were sent and 841 participants responded voluntarily (17% response rate). The dissemination of the survey complied with all ethical principles of informed consent, anonymity and confidentiality. Neither rewards nor any other incentives were offered. Inclusion criteria included the following: being older than 18 years of age; being a Portuguese native speaker from Portugal; and being currently professionally active, whether employed, self-employed or a working student. Ethical approval for this study was granted by the Ethics Committee of the University of Beira Interior, Portugal (code CE-UBI-PJ-2020-088).

### 2.3. Data Analysis

Descriptive statistics were performed to describe the sample (mean, standard deviation, frequencies, and percentages). Data normality was evaluated and confirmed by the Kolmogorov–Smirnov test. To evaluate whether there were differences between comparison groups, Student’s t-distribution tests and one-way ANOVAs were conducted. To assess the association between the variables, Pearson correlation coefficients were measured. Hierarchical multiple regression analyses were performed. Finally, a mediation regression model was used to test the hypothesized causal chain in which burnout was a mediator that explains the underlying mechanism of the relationships between WRQoL and mental health symptoms. To avoid Type I errors, Bonferroni correction tests were run. To measure multicollinearity, we used the variance inflation factor (VIF = 1), which indicated that the variables were not correlated. All statistical procedures were conducted using the Statistical Package for Social Sciences (SPSS version 27, IBM, Armonk, NY, USA) and PROCESS Procedure for SPSS (version 3.5.3, Calgary, Canada).

## 3. Results

Participants

Eight-hundred and forty-one Portuguese active workers between 18 and 67 years of age participated in this study (Mean = 37.23; SD = 11.99). The majority of participants were women (62.5%), single (45.6%), from urban environments (73%), and of middle socioeconomic status (68.3%). Regarding work-related characteristics, the majority of participants were employed (74.6%), did not perform shift work (79.8%), worked for private organizations (54.8%), and worked mostly in the tertiary sector of economic activity (83.6%). The weekly workload in hours was relatively high (Mean = 36.20; SD = 11.89). Table 1 presents the sample’s sociodemographic characteristics in further detail.

Table 2 and Table 3 show the results for participants’ levels of burnout, as well as overall WRQoL and mental health symptoms, by gender and by shift work. Participants’ overall burnout, WRQoL and perceptions of mental health symptoms were moderate; but when analyzing differences by gender, statistically significant differences were found for all variables, except mental distance and depression symptoms (*p* < 0.05), indicating that women participants scored higher in burnout and mental health symptoms and lower in overall WRQoL than men. When analyzing differences by shift work, significant differences were only found for mental health symptoms, showing that participants who worked in shifts presented higher scores.

A correlation matrix was created using all variables to assess the levels of association among burnout, WRQoL and mental health symptoms. As displayed in Table 4, significant correlations were found for all variables.

We also carried out a hierarchical linear regression analysis to assess the effects of age, gender, workload, shift work, WRQoL and burnout on mental health symptoms in the sample. The variables “age”, “gender”, “workload” and “shift” were added in the first block (Model I). “WRQoL” was added in the second block (Model II). All dimensions of “burnout” were added in the third block (Model III). The first block of analysis explained 5% of the variance in mental health symptoms, while the second block explained 17%. When burnout variables were added, the model explained 56% of the variance in mental health symptoms. Furthermore, as shown in Table 5, strong predictors of mental health symptoms in Model III were age, shift work, WRQoL, exhaustion, and cognitive impairment.

**Figure 2:**
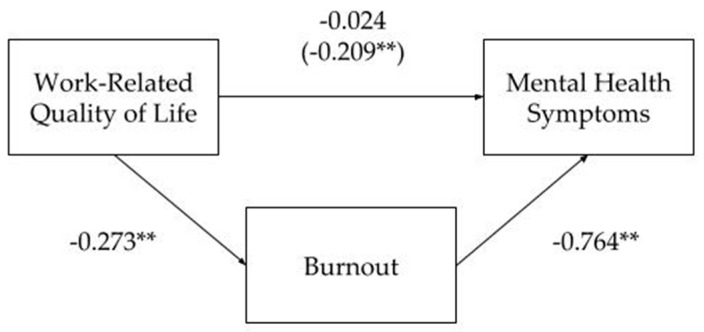
Standardized regression coefficients for the relationship between Work-Related Quality of Life and Mental Health Symptoms as mediated by Burnout. The standardized indirect effect was statistically significant. (** *p* < *0*.001).

Finally, a mediation analysis was performed to assess the mediating effect of burnout on the association between “Overall WRQoL”, which was used as the independent variable, and “Overall Mental Health Symptoms”, which was used as the dependent variable. We used a simple mediation model, with one independent variable (Overall WRQoL), one mediator (Burnout), and one outcome variable (Overall Mental Health Symptoms), providing information to investigate mediation by estimating regression equations. The choice of burnout as a mediator emerged from conceptual theory, which was based on prior research that provided information about the relationship between burnout as a potential mediator of mental health. The indirect effect was calculated by multiplying the path from the independent variable to the mediator variable by the path from the mediator variable to the dependent variable. Figure 2 shows that the standardized regression coefficient between WRQoL and mental health symptoms was statistically significant, as was the standardized regression coefficient between burnout and mental health symptoms. The standardized indirect effect (−0.273) × (−0.764) = −0.209 was significant in a Sobel test of mediation (Z = 11.943; *p* < 0.001). Thus, the indirect effect was statistically significant.

## 4. Discussion

The current research sought to analyze the mediating effect of burnout on the WRQoL-mental health symptoms relationship. Results show that WRQoL exerts an effect on mental health symptoms through burnout. In other words, when burnout is considered, higher WRQoL no longer has a significant direct effect on reducing mental health symptoms, but its influence can be observed indirectly, reducing burnout, which in turn lowers the prevalence of anxiety, depression and somatization symptoms. More specifically, the results suggest that the burnout dimensions of exhaustion and cognitive impairment are key in understanding the reduction of these mental health symptoms. In addition, our results show that WRQoL is a significant negative predictor of burnout, which corroborates previous research findings [21,41,42]. Moreover, the fact that burnout is a significant positive predictor of mental health symptoms contributes to a growing body of evidence that connects burnout to mental health outcomes such as increased anxiety and depression [37,43,44].

From a practical standpoint, the results suggest that organizations seeking to improve workers’ mental health (i.e., reducing their burnout, anxiety, depression and somatization symptoms) should work on employees’ WRQoL. Considering the multifaceted nature of the construct, this means working on improving job and career satisfaction, general well-being, and opportunities for successful home–work balance, while also reducing stress at work, giving workers adequate autonomy and control at work, and improving working conditions.

The results regarding gender and shift work show that women and those who work in shifts report a higher prevalence of burnout and mental health symptoms, and lower WRQoL. These findings are unsurprising and were expected, given that gender disparities already exist in Western societies and in Portuguese society in particular [45,46,47] and that gender inequality in organizational contexts was already widely highlighted by prominent labor organizations [48,49]. These findings are in line with the majority of studies that have shown that women and workers who work in shifts are more negatively affected in these dimensions, most likely because of increased difficulties in managing the disproportionate share of domestic work and family care burdens [50,51,52] placed upon them, which can negatively influence their feelings of stress and exhaustion [53]. In this regard, our results contribute to a broader analysis of the gender distribution of work in Portugal, where women have historically been more responsible for domestic chores and family care and often face the need to balance those tasks with paid employment [54].

Findings from this study need to be seen in the context of its limitations. The cross-sectional research method hinders a broader understanding of the dynamics of the variables across time. Moreover, while convenient, the sample was non-representative, which makes it unclear to what extent we can generalize our findings. The sample is mostly comprised of tertiary sector workers (commerce, services and education workers) and non-shift workers, which limits the interpretation of our results to other, underrepresented sectors. Although most of the Portuguese working population operates in the tertiary sector, its overrepresentation in our sample might be related to another limitation; that is, the fact that only those with access to the internet were able to answer. Additionally, the fact that the survey did not focus on a specific professional group, such as physicians [55] or pharmacists [56], but on a wide range of different occupations, hinders a more precise discussion of the practical implications. Finally, given that the questionnaire was made available online, the study’s characteristics raise questions regarding the possible influence of selection bias.

In order to address these limitations, the researchers suggest that future studies utilize samples that are representative of the Portuguese populace as a whole. More representative samples would allow future research to provide more generalizable and accurate estimates of results. Moreover, future longitudinal studies could also enhance the understanding of the mediating effect of burnout on the relationship between WRQoL and mental health symptoms over time.

## 5. Conclusions

Our results are similar to those of previous research [57] and have important practical implications, suggesting that organizations should be more attentive to certain groups of workers at a higher risk of developing mental health problems in the presence of occupational hazards, thus preventing negative organizational outcomes.

The findings are useful for professionals and managers who work in the field of occupational health in identifying variables affecting burnout, WRQoL and mental health symptoms. Therefore, in order to provide better occupational health services, occupational health professionals and human resources management teams must provide adequate occupational conditions, paying special attention to the variables under study, in order to positively influence workers’ well-being, and reduce work-life conflicts.

## Figures and Tables

**Table 1:** Sociodemographic characteristics of the participants (*N* = 841; M*_age_* = 37.23; SD = 11.99; M*_workload_* = 36.20; SD = 11.89).

		*N*	*%*
Gender	Women	526	62.5
	Men	315	37.5
Marital Status	Single	383	45.6
	Married	271	32.2
	De facto union	121	14.4
	Divorced/Separated	59	7
	Widower	7	0.8
Educational Attainment	Up to 12 years of school	304	36.2
	Undergraduate degree	240	28.5
	Postgraduate degree	297	35.3
Place of residence	Small rural	131	15.6
	Big rural	96	11.4
	Small urban	383	45.5
	Big urban	231	27.5
Socioeconomic Status	Low	33	3.9
	Middle-Low	178	21
	Middle	490	58.3
	Middle-High	137	16.3
	High	4	0.5
Professional Status	Working student	98	11.7
	Self-employed	115	13.7
	Employed	628	74.6
Shift work	Yes	170	20.2
	No	671	79.8
Nature of the Organization	Public	316	37.6
	Private	461	54.8
	Other	64	7.6
Sector of Activity	Primary (agriculture, feedstock, fishing, etc.)	32	3.8
	Secondary (industry)	106	12.6
	Tertiary (commerce, services, education, etc.)	703	83.6

**Table 2:** Results for Burnout, Overall Work-Related Quality of Life and Mental Health Symptoms by Gender.

		Gender	M(SD)	t(df)	*p*
Burnout	Exhaustion	Men	2.47(0.68)	−4.847(756)	0.000 **
		Women	2.70(0.61)		
	Mental distance	Men	1.81(0.75)	−363(752)	0.717
		Women	1.83(0.69)		
	Emotional impairment	Men	2.21(0.70)	−2.169(755)	0.030 *
		Women	2.31(0.60)		
	Cognitive impairment	Men	2.15(0.72)	−4.173(761)	0.000 **
		Women	2.36(0.67)		
	Overall Burnout	Men	2.16(0.60)	−3.505(763)	0.000 **
		Women	2.30(0.51)		
Overall WRQoL		Men	3.61(0.97)	2.390(749)	0.017 **
		Women	3.44(0.96)		
Mental Health	Somatization Symptoms	Men	0.44(0.56)	−3.882(698)	0.000 **
		Women	0.62(0.62)		
	Depression Symptoms	Men	0.78(0.84)	−1.759(696)	0.079
		Women	0.88(0.75)		
	Anxiety Symptoms	Men	0.75(0.67)	−3.974(697)	0.000 **
		Women	0.96(0.70)		
	Overall Mental Health Symptoms	Men	0.66(0.61)	−3.484(698)	0.001 *
		Women	0.82(0.61)		

Note: * *p* < 0.05; ** *p* < 0.001.

**Table 3:** Results for Burnout, Overall Work-Related Quality of Life and Mental Health Symptoms by Shift Work.

		Shift Work	M(SD)	t(df)	*p*
Burnout	Exhaustion	Yes	2.67(0.70)	1.132(773)	0.258
		No	2.60(0.64)		
	Mental distance	Yes	1.89(0.81)	1.098(769)	0.273
		No	1.82(0.79)		
	Emotional impairment	Yes	2.27(0.69)	−0.069(772)	0.945
		No	2.27(0.63)		
	Cognitive impairment	Yes	2.34(0.79)	1.014(778)	0.311
		No	2.27(0.68)		
	Overall Burnout	Yes	2.29(0.61)	1.031(780)	0.303
		No	2.24(0.54)		
Overall WRQoL		Yes	3.38(1.02)	−1.688(766)	0.092
		No	3.53(0.95)		
Mental Health	Somatization Symptoms	Yes	0.74(0.70)	4.131(708)	0.000 **
		No	0.51(0.56)		
	Depression Symptoms	Yes	1.01(0.85)	2.878(706)	0.004 *
		No	0.80(0.77)		
	Anxiety Symptoms	Yes	1.06(0.81)	3.544(707)	0.000 **
		No	0.84(0.66)		
	Overall Mental Health Symptoms	Yes	0.94(0.72)	3.905(708)	0.000 **
		No	0.71(0.58)		

Note: * *p* < 0.05; ** *p* < 0.001.

**Table 4:** Correlation matrix.

	1	2	3	4	5	6	7	8	9	10
1-Exhaustion										
2-Mental distance	0.545 **									
3-Emotional impairment	0.588 **	0.505 **								
4-Cognitive impairment	0.602 **	0.491 **	0.562 **							
5-Overall Burnout	0.836 **	0.791 **	0.807 **	0.822 **						
6-Overall WRQoL	−0.369 **	−0.529 **	−0.286 **	−0.282 **	−0.450 **					
7-Somatization Symptoms	0.464 **	0.293 **	0.362 **	0.460 **	0.483 **	−0.188 **				
8-Depression Symptoms	0.589 **	0.562 **	0.521 **	0.642 **	0.711 **	−0.428 **	0.580 **			
9-Anxiety Symptoms	0.564 **	0.413 **	0.495 **	0.655 **	0.653 **	−0.309 **	0.700 **	0.766 **		
10-Overall Mental Health Symptoms	0.610 **	0.483 **	0.525 **	0.662 **	0.700 **	−0.359 **	0.833 **	0.900 **	0.928 **	

Note: ** *p* < 0.001.

**Table 5:** Hierarchical multiple regression analysis predicting overall Mental Health Symptoms.

	Model I			Model II			Model III		
	*B*	*SE B*	*β*	*B*	*SE B*	*β*	*B*	*SE B*	*β*
Age	−0.006	0.002	−0.112 *	−0.005	0.002	−0.091 *	−0.004	0.001	−0.074 *
Gender	0.172	0.048	0.136 **	0.142	0.046	0.112 *	0.031	0.034	0.025
Workload	0.001	0.002	0.014	0.000	0.002	0.003	−0.001	0.001	−0.017
Shift	−0.195	0.061	−0.124 *	−0.179	0.058	−0.115 *	−0.147	0.042	−0.094 *
WRQoL				−0.227	0.024	−0.349 **	−0.046	0.021	−0.071 *
Exhaustion							0.239	0.036	0.253 **
Mental distance							0.049	0.031	0.057
Emotional impairment							0.056	0.035	0.059
Cognitive impairment							0.372	0.032	0.427 **
$R^{2}$			0.054			0.174			0.560
F			9.059 **			26.860 **			89.399 **

Note: * *p* < 0.05; ** *p* < 0.001.

## Data Availability

The data presented in this study are available upon request.
